# New therapies to treat inherited cardiomyopathies are on the way

**DOI:** 10.1007/s12471-021-01657-5

**Published:** 2022-01-20

**Authors:** P. Vlasman, J. van der Velden

**Affiliations:** Amsterdam Cardiovascular Sciences, Amsterdam University Medical Centres, location VU University Medical Centre, Vrije Universiteit Amsterdam, Amsterdam, The Netherlands

In the fifties of the previous century, several reports appeared on asymmetric hypertrophy of the heart in young adults. This discovery turned out to be a familial cardiac disease termed hypertrophic cardiomyopathy (HCM) and was followed by the identification of the genetic cause in 1989: a mutation in the gene encoding the sarcomeric protein myosin heavy chain. Since then, multiple pathogenic gene variants have been identified in the building blocks of cardiac muscle cells that regulate contraction and relaxation of the heart. While sarcomere gene mutations cause both HCM and dilated cardiomyopathy (DCM), mutations in the calcium handling protein phospholamban (PLN) cause DCM [1, 2]. Scientists in the Netherlands have been leading in the research on a Dutch PLN founder mutation that causes DCM and restrictive cardiomyopathy [3].

In the current issue of the *Netherlands Heart Journal*, a multicentre team describes the study design and the baseline characteristics of participants of i‑PHORECAST. The aim of this intervention study is to test the efficacy of eplerenone, a mineralocorticoid receptor antagonist with established antifibrotic effects, in preventing or delaying the onset of cardiomyopathy in asymptomatic, preclinical individuals who carry a PLN founder mutation [4]. This multicentre trial in individuals with the exact same mutation has great potential, as studies in cell systems and animals have shown that mutation-mediated pathomechanisms depend on the affected gene and may even depend on mutation location. As such, to establish the beneficial effects of a therapy to prevent mutation-mediated cardiac remodelling and dysfunction, individuals with a founder mutation represent a unique cohort in which to perform a clinical drug study. Moreover, a clear definition of the primary end point is crucial to establish efficacy of the drug intervention, in particular in a disease that has a relatively slow disease progression and, compared with heart failure patients, has low mortality rates.

The study design of i‑PHORECAST is powerful and includes excellent cardiac phenotyping with cardiac magnetic resonance, electrocardiography, Holter monitoring and exercise testing, which allows for multiparametric assessment of disease progression in a well-genotyped participant group. A major challenge was to enrol a sufficient number of participants, and enrolment was closed when 84 persons entered the trial instead of the 150 that was aimed for. It took about four years to include these 84 individuals, which illustrates the challenge to perform clinical intervention studies in individuals who carry a mutation but are not ill.

Inclusion of non-symptomatic gene carriers requires a thorough investment in the creation of a patient-centred trial design. Informing patients about the goals of the trial and gathering their inputs are critical success factors, especially when non-symptomatic individuals are asked to undergo clinical examinations or take drugs. Historically, clinical trials have been successful in enrolling Caucasian male adults, whereas women and minorities are less likely to participate as they have more logistical or linguistic burdens or special needs such as childcare, transportation and loss of pay. By engaging directly with patients with inherited cardiomyopathies and their family members during the recruitment and enrolment process, barriers such as lack of knowledge and logistic hurdles involving transport and childcare may be solved and patient dropout could perhaps be prevented.

Furthermore, for longitudinal studies, a repeated informed consent is highly recommended. Future studies aimed to assess the preventive effect of new drugs at a preclinical stage in inherited cardiomyopathies, can be further strengthened by expanding the study design to a cross-over drug intervention, although this would result in an even longer treatment period for participating individuals and additional clinical examinations.

While genetic cardiomyopathies have a relatively young history, multiple newly developed drugs are moving to clinical practice [1, 2, 5]. These drugs may have the potential to not only improve cardiac function and reverse symptoms in patients with advanced disease but may also exert preventive effects on cardiac remodelling and prevent or delay disease onset. The collaborative national and international efforts to build registries, biobanks and communication platforms to inform patients and their relatives will aid well-designed clinical trials in testing these new drugs. It is essential that clinicians and researchers team up with patient representatives and jointly discuss recent advances in research and the pros and cons of performing certain clinical studies, as well as decide which trial has the highest chance of success.

We look forward to the final results of i‑PHORECAST. For now, the medical community can already build on the lessons learned from this study to improve future clinical trials.
